# Vitamin D Deficiency in Childhood Cancer Survivors: Results from Southern Thailand

**DOI:** 10.3390/nu15061328

**Published:** 2023-03-08

**Authors:** Sirinthip Kittivisuit, Pornpun Sripornsawan, Natsaruth Songthawee, Shevachut Chavananon, Umaporn Yam-ubon, Edward B. McNeil, Somchit Jaruratanasirikul, Thirachit Chotsampancharoen

**Affiliations:** 1 Department of Pediatrics, Faculty of Medicine, Prince of Songkla University, Hat Yai 90110, Thailand; 2 Epidemiology Unit, Faculty of Medicine, Prince of Songkla University, Hat Yai 90110, Thailand

**Keywords:** childhood cancer survivors, cancer survivorship, late effects, risk factor, vitamin D deficiency

## Abstract

**Simple Summary:**

Vitamin D deficiency, defined as a total 25-hydroxyvitamin D level of less than 20 ng/mL, is one of the major global health problems in children. There are few studies on vitamin D deficiency in childhood cancer survivors (CCSs), especially in tropical countries. Our study enrolled a total of 206 CCSs between January 2021 and March 2022 to evaluate the prevalence and risk factors for vitamin D deficiency. We found that CCSs had a high prevalence of vitamin D deficiency (35.9%), even in tropical areas such as southern Thailand. Female gender, obesity, lack of outdoor activities, and lower dietary dairy intake were independent risk factors for vitamin D deficiency. We believe that our results will be of benefit to clinicians who take care of CCSs. Regular screening should be established in long-term CCS care to identify those who are at risk of vitamin D deficiency and should be receiving appropriate supplementation.

**Abstract:**

There is limited information on vitamin D deficiency among childhood cancer survivors (CSS), especially in tropical countries. The aims of this study are to determine the prevalence and risk factors for vitamin D deficiency in CCSs. This study was conducted at the long-term follow-up clinic for CCSs at Prince of Songkla University, Songkhla, Thailand. All CCSs who were followed up between January 2021 and March 2022 were enrolled. Demographic data, dietary dairy intake, average weekly duration of outdoor activities, total 25-hydroxyvitamin D [25(OH)D] levels, parathyroid hormone levels, and blood chemistry were collected. A total of 206 CCSs with a mean age at follow-up of 10.8 ± 4.7 years were included. The prevalence of vitamin D deficiency was 35.9%. Female gender (odds ratio (OR): 2.11, 95% CI: 1.08–4.13), obesity (OR: 2.01, 95% CI: 1.00–4.04), lack of outdoor activities (OR: 4.14, 95% CI: 2.08–8.21), and lower dietary dairy intake (OR: 0.59, 95% CI: 0.44–0.80) were independent risk factors for vitamin D deficiency. Vitamin D deficiency was common in CCSs and associated with female gender, obesity, lack of outdoor activities, and lower dietary dairy intake. Regular 25(OH)D screening should be established in long-term care to identify those who require vitamin D supplements.

## 1. Introduction

In recent years there have been increasing numbers of childhood cancer survivors (CCSs) as a result of advancements in cancer therapy [1]. Surveillance of long-term health effects, including bone health problems, is important in this population [2,3,4]. Although vitamin D is essential for maintaining bone health, vitamin D deficiency, defined as a total 25-hydroxyvitamin D [25(OH)D] level of less than 20 ng/mL, is one of the major global health problems in children, with a prevalence ranging from 7% to 95% in general populations [5,6,7]. Previous studies that assessed the vitamin D status of healthy children in Thailand reported that the prevalence of vitamin D deficiency (25(OH)D < 20 ng/mL) was 19.5–33.4% [8,9,10,11]. Female gender, older age, obesity, limited dietary dairy intake, limited sun exposure, geographic region, and seasonal period have been reported as risk factors for vitamin D deficiency [8,9,10,11,12,13,14,15]. In addition to these potential risks, CCSs have increased risks for vitamin D deficiency, including restrictions on outdoor activities and exposure to steroids, chemotherapy, and/or radiation. Therefore, screening for vitamin D deficiency in CSS is necessary. A meta-analysis that included 19 studies reported that the median prevalence of vitamin D deficiency in pediatric cancer patients was 14%, with a range of 0% to 61.5% [16]. However, these studies concentrated on specific cancer diagnoses and were heterogeneous in the definitions of vitamin D deficiency and the time point of vitamin D measurement (at diagnosis, during therapy, and on completion of therapy). There are few studies on vitamin D deficiency in CCSs. Previous studies have reported the prevalence of vitamin D deficiency in CCSs, varying from 14% to 48% [17,18,19,20,21]. Considering that there is limited information on vitamin D deficiency among CCSs in tropical regions with an abundance of sunlight, such as southern Thailand, this study aims to identify the prevalence and clinical risk factors of vitamin D deficiency in CCSs in southern Thailand.

## 2. Materials and Methods

This cross-sectional study included all CCSs who were followed up at the long-term follow-up clinic for childhood cancer, Department of Pediatrics, Faculty of Medicine, Prince of Songkla University, Songkhla, southern Thailand. Our hospital is a major tertiary healthcare institution and referral center in southern Thailand. Songkhla is located at latitude 7.20° N and longitude 100.60° E and has a tropical climate, with only dry and rainy seasons. Although there are seasonal variations of ultraviolet radiation, there is plenty of sunshine all year [22]. The study was approved by the Ethics Committee, Faculty of Medicine, Prince of Songkla University. Written informed consent and written assent were obtained from all parents and participants.

All CCSs who were followed up between January 2021 and March 2022 were enrolled. Each participant had completed therapy and was in remission. The cancer diagnoses were categorized into 3 groups: leukemia/lymphoma (acute lymphoblastic leukemia, acute myeloid leukemia, Hodgkin lymphoma, non-Hodgkin lymphoma), solid tumor (Ewing sarcoma, rhabdomyosarcoma, osteosarcoma, neuroblastoma, hepatoblastoma, Wilm tumor, retinoblastoma, germ cell tumor, Langerhans cell histiocytosis), and brain tumor (medulloblastoma, astrocytoma, primitive neuro-ectodermal tumor, germ cell tumor). Participants who were receiving vitamin D supplements were excluded from the study. The medical records of all enrolled participants were retrospectively reviewed for cancer diagnosis and treatment. The information recorded for each participant at the follow-up visit included demographic characteristics (age, weight, height, body mass index, pubertal status), amount of dietary dairy intake, average weekly duration of outdoor activities, 25(OH)D, parathyroid hormone (PTH) level, and blood chemistry readings. For dietary dairy intake, only milk consumption was recorded as milliliters per week. The duration of outdoor activities in which the participants were exposed to sunlight was recorded in hours per week.

### 2.1. Anthropometric Data Collection

Body weight was measured using an electronic scale, with the participants wearing only light clothing and without shoes. Height was measured with a stadiometer. Body mass index (BMI) was calculated by dividing weight in kilograms by height in meters squared and then converted to a BMI percentile according to the Centers for Disease Control and Prevention growth charts for age/sex-adjusted children and teens aged 2 through 19 years [23]. A BMI of less than the 5th percentile was defined as underweight, the 5th through 84th percentiles as a healthy weight, and the 85th through 99th percentiles as overweight or obese. Pubertal development for each participant was determined according to the Tanner staging system. For females, prepubertal status was defined as Tanner stage I breast development, and for males, a testicular volume of less than 4 mL [24].

### 2.2. Vitamin D Levels and Biochemistry Analyses

Total serum 25(OH)D is the major circulating form of vitamin D and, thus, the best indicator for measuring vitamin D status. Total serum 25(OH)D levels were measured for all participants by chemiluminescent immunoassay using the LIAISON analyzer (DiaSorin, Stillwater, MN, USA) and were recorded in nanograms per milliliter (ng/mL). The inter-assay coefficients of variation for the serum 25(OH)D levels were in the range of 8.3–9.7%. Following the 2011 Endocrine Society Guidelines, vitamin D levels of <20 ng/mL, 21–29 ng/mL, and ≥30 ng/mL were defined as deficient, insufficient, and sufficient, respectively [5]. Parathyroid hormone levels were measured by electrochemiluminescent immunoassay using the Elecsys PTH STAT e 411 analyzer (Roche Diagnostics, Mannheim, Germany). The inter-assay coefficients of variation for the serum PTH levels were in the range of 2.7–3.4%. Other biochemistry values were measured using the Alinity analyzer (Abbott, Deerfield, IL, USA). Estimated glomerular filtration rate (eGFR) was used to determine kidney function by calculating creatinine clearance using the original Schwartz formula with a modified Jafe assay and a modified Schwartz formula with enzymatic creatinine results [25,26]. An eGFR was considered to have decreased if it fell below 90 mL/min/1.73 m^2^.

### 2.3. Statistical Analysis

Descriptive statistics are presented using mean and standard deviation (SD) or median and interquartile range (IQR) for continuous variables, as appropriate, and frequency with percentage for categorical variables. Variables associated with vitamin D deficiency were analyzed using the chi-square test or Fisher’s exact test for categorical variables and Student’s *t*-test or the rank-sum test for continuous variables, as appropriate. Variables having a *p*-value of less than 0.1 from the univariate analysis were included in the initial multivariate logistic regression model for the assessment of independent risk factors. The final model was selected using a stepwise backward elimination method based on the likelihood ratio test. The risk factors for vitamin D deficiency are presented as adjusted odds ratios (ORs) with 95% confidence intervals (CIs). A *p*-value less than 0.05 was considered significant.

## 3. Results

### 3.1. Baseline Characteristics of the Study Participants

A total of 206 CCSs were included in the study. None of the participants had received or were receiving vitamin D supplements. Most of the participants were male (59.2%). The distribution of cancer diagnoses and treatment of the 206 study participants are presented in Table 1. The most common diagnoses were leukemia or lymphoma (49.0%). Within the leukemia or lymphoma group, acute lymphoblastic leukemia was the most common type (61.4%). Solid tumors were diagnosed in 40.3% of the total study population. The three most frequent diagnoses in the solid tumor group were Langerhans cell histiocytosis (16.9%), retinoblastoma (15.7%), and germ cell tumor (14.5%). Brain tumors were the least common cancer diagnoses (10.7%), and medulloblastoma was the most common brain tumor (40.9%). Approximately half of the participants received intrathecal chemotherapy and steroids. One-fourth of the participants were exposed to radiation during treatment.

### 3.2. Demographic Characteristics at the Follow-Up Visit

The mean age at follow-up was 10.8 ± 4.7 years. The median (IQR) time from the end of cancer therapy to the follow-up visit was 2.3 (1.0–3.9) years. Most of the participants were classified as having a normal BMI (55.8%), followed by obese (31.6%) and underweight (12.6%). The proportion of participants in the prepubertal and pubertal stages was comparable. Most of the participants (68%) spent their time outdoors, with a median duration of 3.0 h per week, and consumed dietary dairy products, with a median of 1250.0 (750.0–2400.0) milliliters per week. The demographic and laboratory characteristics of the study population are presented in Table 2.

### 3.3. Vitamin D Status and Biochemistry Measurements

**Figure 1:**
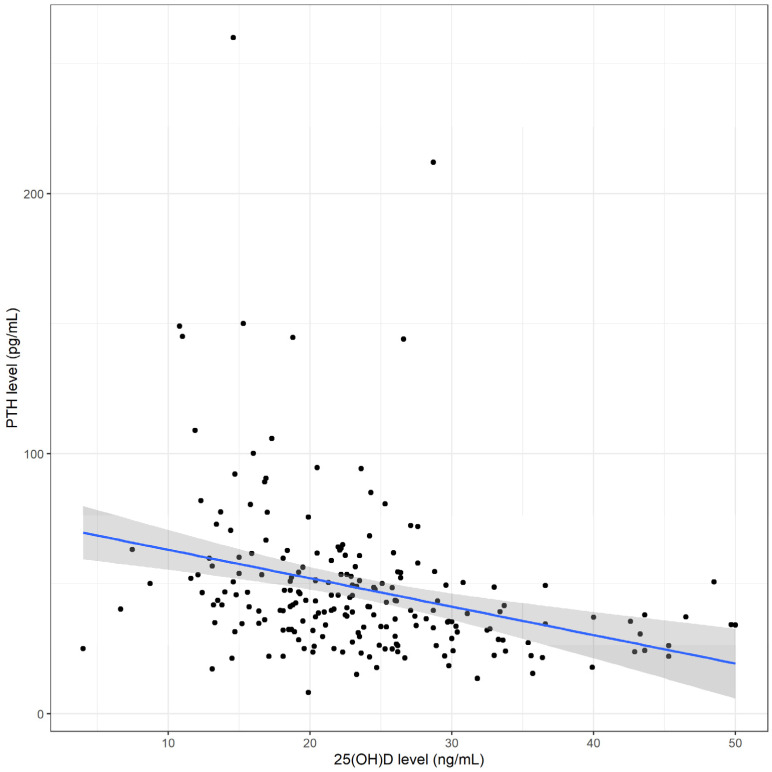
Correlation between serum total 25-hydroxyvitamin D [25(OH)D] levels and serum parathyroid hormone (PTH) levels. The blue line represents the fitted regression line and the grey shadow the 95% confidence interval.

Overall, the mean (SD) vitamin D level was 10.8 (4.7) ng/mL. Of the 206 children, 74 (35.9%) had vitamin D deficiency, 96 (46.6%) had vitamin D insufficiency, and 36 (17.5%) had vitamin D sufficiency. Among the 74 children who had vitamin D deficiency, 8 (10.8%) were defined as having severe deficiency (<12 ng/mL). Serum 25(OH)D levels were significantly inversely correlated with serum PTH levels (r = −0.3, *p* < 0.001) (Figure 1). The median (IQR) PTH level was 41.2 (32.0–53.8) pg/mL. Hyperparathyroidism (PTH level >65 pg/mL) was identified in 25.7% (19/74) of vitamin-D-deficient children, 9.4% (9/96) of vitamin-D-insufficient children, and none of the vitamin-D-sufficient children.

### 3.4. Risk Factors for Vitamin D Deficiency

The participants were classified into two groups according to vitamin D status: those with vitamin D deficiency (serum 25(OH)D levels <20 ng/mL; *n* = 74) and those without vitamin D deficiency (serum 25(OH)D levels >20 ng/mL; *n* = 132). On univariate analysis, the mean age at follow-up among the children who had vitamin D deficiency was significantly higher than in those who did not (12.5 vs. 9.9 years, respectively, *p* < 0.001). Vitamin D deficiency in females was significantly more frequent than in males (52.7 vs. 47.3%, *p* = 0.014). Children who had vitamin D deficiency had significantly higher weight, height, and BMI than those who did not have vitamin D deficiency (*p* < 0.001). Vitamin D deficiency was more frequent in children who were obese. (41.9 vs. 25.8%, respectively, *p* = 0.025). Vitamin D deficiency was diagnosed in children who had already entered puberty significantly more frequently than in prepubertal children (67.6 vs. 38.6%, respectively, *p* < 0.001). Children who did not engage in outdoor activities were significantly more likely to have vitamin D deficiency compared with those who did (56.8 vs. 18.2%, respectively, *p* < 0.001). The median amount of dietary dairy intake per week was significantly lower among the children with vitamin D deficiency than in the other group (1000.0 vs. 1500.0 mL, respectively, *p* < 0.001). The children with vitamin D deficiency had significantly higher PTH levels and lower serum calcium levels compared with those who did not (*p* < 0.001 and *p* = 0.009, respectively). However, other biochemistry tests associated with vitamin D status, serum phosphorus, and alkaline phosphatase levels were not significantly different. Other variables, including cancer diagnosis, treatment, follow-up time, alanine aminotransferase, albumin, hemoglobin, serum iron, total iron binding capacity, transferrin saturation, ferritin, zinc, and estimated glomerular filtration rate, were not significantly different between children with and without vitamin D deficiency. A comparison of the demographic and laboratory characteristics between the 74 children who had vitamin D deficiency and the 132 children who did not is presented in Table 2.

On multivariate analysis, the independent risk factors for vitamin D deficiency are shown in Table 3. There were four risk factors that were statistically significant for vitamin D deficiency: female gender, obesity, lack of outdoor activities, and lower dietary dairy intake. Females had an odds ratio of 2.11 (95% CI: 1.08–4.13) for vitamin D deficiency compared to males (*p* = 0.029). In comparison to those who were not obese, participants with obesity had an odds ratio of 2.01 (95% CI: 1.00–4.04) for vitamin D deficiency (*p* = 0.05). Participants who did not engage in outdoor activities had an odds ratio of 4.14 (95% CI: 2.08–8.21) in comparison to those who did (*p* < 0.001). Lower dietary dairy intake was a significant risk factor for vitamin D deficiency, with an odds ratio of 0.59 (95% CI: 0.44–0.80) (*p* < 0.001).

## 4. Discussion

Our study included a large and diverse population of 206 childhood cancer survivors. We found that the prevalence of vitamin D deficiency and insufficiency in our study were 35.9% and 46.6%, respectively. Our prevalence was higher than that reported in healthy Thai children [8,9,10,11]. Similarly, Sinha et al. reported that children with cancer had vitamin D levels of less than 10 ng/mL more frequently than a healthy control group [27]. Gunes et al. found that the vitamin D levels of 70 CCSs were lower than in normal controls [28]. In contrast, Simmons et al. found that the prevalence of vitamin D deficiency among 78 survivors of acute lymphoblastic leukemia (ALL) was similar to the reported prevalence in the general pediatric population [29]. Even though it has been observed that CCSs tended to have a higher prevalence of vitamin D deficiency than the general population, the causal relationship between vitamin D deficiency and CCSs remains unclear. It has been proposed that CCSs may be more susceptible to vitamin D deficiency due to several circumstances, including the impact of the disease, treatment-related factors (exposure to steroids, chemotherapy, and/or radiation), inadequate nutritional intake, and restrictions on outdoor activities [12,20,27,29].

There are few studies of vitamin D deficiency in CCSs. Previous studies have reported rates of prevalence varying from 14% to 48% [17,18,19,20,21]. Rosen et al. retrospectively reviewed 201 CCSs and reported that 14% had vitamin D deficiency [17]. Similarly, Esbenshade et al. reported that 16% of 171 CCSs in their study had vitamin D deficiency [18]. Vitamin D deficiency was more prevalent among CCSs in studies by Bhandari et al., Choudhary et al., and Modan-Moses et al., with rates of 24%, 29%, and 48%, respectively [19,20,21]. However, the majority of these studies were conducted in regions of temperate climates. Our study, conducted in a region of tropical climate, located at the latitude of 7.20° N, with plenty of sunlight, found that the prevalence of vitamin D deficiency was 35.9%, which was within the upper range of previous studies in CCSs. The varying prevalence of vitamin D deficiency observed in the literature might be, at least partly, accounted for by geographic differences.

When focusing on subgroups of specific cancer diagnoses, a few studies have evaluated vitamin D deficiency in survivors of leukemia. Simmons et al., using a different definition of vitamin D deficiency, reported that 11.5% of 78 ALL survivors had serum 25(OH)D levels of less than 15 ng/mL [29]. Delvin et al. investigated 251 ALL survivors and found that 32.7% had vitamin D deficiency [30]. A study by Schündeln et al. reported that 71.8% of 124 ALL survivors had vitamin D deficiency [31]. Our study, which included a total of 101 survivors of leukemia and lymphoma, found that the prevalence of vitamin D deficiency in this subgroup was 38.6% (39/101), which was within the range of those previous studies. There are only a limited number of studies that have investigated vitamin D deficiency in survivors of solid or brain tumors. Bilariki et al. reported that 61.5% of 52 survivors of solid or brain tumors in their study had vitamin D deficiency [32]. Our study found a lower prevalence of vitamin D deficiency in survivors of solid or brain tumors of 32.5% (27/83) and 36.4% (8/22), respectively. The differing prevalence may be due, at least partly, to the different methods of measuring serum 25(OH)D levels, the threshold for diagnosing vitamin D deficiency, and differences in associated factors affecting the vitamin D status, including geographic area, seasonality, sun exposure habits, skin pigmentation, and consumption of vitamin D either in natural or fortified food sources. Apart from infant formula, there are no regulations specifying food to be fortified with vitamin D (i.e., cereals, yogurts, cheeses, butter, and margarine) under the law in Thailand, as they do in some other countries. Furthermore, these foods are not commonly consumed by the majority of Thai children.

Female gender, older age, obesity, limited dietary dairy intake, limited sun exposure, geographic region, and seasonal period have been reported as risk factors for vitamin D deficiency [8,9,10,11,12,13,14,15]. Similarly, our study found that female gender, obesity, lack of outdoor activities, and lower dietary dairy intake were risk factors for vitamin D deficiency. On the other hand, older age did not appear to be a significant risk factor for vitamin D deficiency in our study. There is still some controversy around the potential risk factors for vitamin D deficiency in the CCS population. Some studies have reported older age to be a significant risk factor for vitamin D deficiency in CCSs [17,18,21,24,27], while other studies did not find this association [19,20]. In our study, although CCSs who had vitamin D deficiency were generally older than those who did not, age was not identified as a risk factor for vitamin D deficiency in the multivariate analysis. We found that the female gender was a potential risk factor for vitamin D deficiency, which was also found in a previous study in a general pediatric population [12] but not in previous studies in CCS populations [17,18,19,20,21,27]. We also found that obesity was associated with vitamin D deficiency, similar to previous studies [18,19]. As a result of the fact that there is no consensus in previous studies on CCSs regarding the potential risk factors for vitamin D deficiency, further multicenter prospective studies involving larger and more diverse CCS populations are necessary to consolidate the risk factors for vitamin D deficiency in CCSs.

We observed an inverse correlation between serum 25(OH)D and PTH levels. Children with vitamin D deficiency exhibited lower serum calcium levels; however, their serum phosphorus and alkaline phosphatase levels were similar to those of children without vitamin D deficiency. These findings could be explained by the effects of PTH, calcium, and phosphate metabolism in the vitamin-D-deficiency state. Although the lower serum calcium was statistically significant, the difference was not considered clinically significant.

There is a limited number of studies that have investigated associations between outdoor activities and dairy intake and CCSs. Our study found that a lack of outdoor activities and a lower dairy intake were risk factors for vitamin D deficiency. Similarly, the amount of sun exposure was associated with higher serum 25(OH)D levels in a study by Modan-Moses et al. [21]. Steroid use has been reported to be associated with vitamin D deficiency [12]; however, exposure to steroids, chemotherapy, or radiation was not identified as a risk factor for vitamin D deficiency in our study.

To the best of our knowledge, this is the first study to evaluate the prevalence of vitamin D deficiency and insufficiency among CCSs in a tropical region. The study was conducted using a cross-sectional design, and the sample size was large in comparison to studies on CCSs. However, our study also had some limitations. First, several statistical comparisons were performed without using multiple testing correction (which is appropriate for an exploratory study); however, these methods may uncover associations that could be spurious, and therefore, this potential limitation should be mentioned. Second, some information was self-reported. Therefore, some errors might have been introduced. Third, this study was performed in a limited geographic area of southern Thailand. The findings should be interpreted in consideration of these points. Fourth, several related variables, including the use of sunscreen and clothing, the diurnal variations of sun exposure, the consumption of other dairy products besides milk, and additional dietary sources of vitamin D, were not evaluated. However, the main food component for Thai children does not consist of cheese or other dairy products. The natural dietary sources of vitamin D include oily fish (sardines, tuna, mackerel, salmon), cod liver oil, egg yolks, and organ meats (liver, kidney), with varying vitamin D content. However, the majority of these foods are not commonly consumed by Thai children. In addition, food preparation, which was also not recorded, can have a significant effect on vitamin D content. Further studies incorporating these variables that may influence vitamin D levels are warranted to confirm our results. Additionally, the effects of vitamin D deficiency on health outcomes, such as decreased bone mineral density and fractures, were not assessed.

## 5. Conclusions

The prevalence of vitamin D deficiency in childhood cancer survivors was one-third of the participants in our study. Female gender, obesity, lack of outdoor activities, and lower dietary dairy intake were significant risk factors for vitamin D deficiency. Although Thailand has no official recommendations for the routine screening of vitamin D levels in CCSs, our findings may contribute to the implementation of risk-based screening to identify children who are at risk of vitamin D deficiency and should be receiving appropriate supplementation.

## Figures and Tables

**Table 1:** Cancer diagnosis and treatment of the 206 study participants.

Variable ^†^	Total (%)
**Cancer Diagnosis**	
Leukemia/lymphoma	101 (49.0)
Solid tumor	83 (40.3)
Brain tumor	22 (10.7)
Leukemia/lymphoma	(*N* = 101)
Acute lymphoblastic leukemia	62 (61.4)
Acute myeloid leukemia	12 (11.9)
Non-Hodgkin lymphoma	17 (16.8)
Hodgkin lymphoma	10 (9.9)
Solid tumor	(*N* = 83)
Ewing sarcoma	7 (8.4)
Rhabdomyosarcoma	6 (7.2)
Osteosarcoma	10 (12.0)
Neuroblastoma	6 (7.2)
Hepatoblastoma	5 (6.0)
Wilm tumor	8 (9.6)
Retinoblastoma	13 (15.7)
Germ cell tumor	14 (16.9)
Langerhans cell histiocytosis	12 (14.5)
Others	2 (2.4)
Brain tumor	(*N* = 22)
Medulloblastoma	9 (40.9)
Astrocytoma	5 (22.7)
Primitive neuro-ectodermal tumor	4 (18.2)
Germ cell tumor	4 (18.2)
**Treatment**	
Intrathecal chemotherapy	
Yes	87 (42.2)
No	119 (57.8)
Steroids	
Yes	101 (49.0)
No	105 (51.0)
Cumulative steroids (mg/m^2^)	6400 (4600–8400)
Surgery	
Yes	94 (45.6)
No	112 (54.4)
Radiation	
Yes	55 (26.7)
No	151 (73.3)
Cumulative radiation dosage (Gray)	46 (30–54)

^†^ Values are expressed as *n* (%) or median (IQR).

**Table 2:** Univariate analysis comparing demographic and clinical characteristics between the 74 childhood cancer survivors who had vitamin D deficiency and the 132 who did not.

Variable ^†^	Total (*N* = 206)	Vitamin D Deficiency (*N* = 74)	No Vitamin D Deficiency (*N* = 132)	*p*-Value
**Demographic and Clinical Characteristics**				
Age at follow-up visit (years)	10.8 ± 4.7	12.5 ± 4.0	9.9 ± 4.8	<0.001
Sex				0.014
Male	122 (59.2)	35 (47.3)	87 (65.9)	
Female	84 (40.8)	39 (52.7)	45 (34.1)	
Diagnosis				0.519
Leukemia/lymphoma	101 (49.0)	39 (52.7)	62 (47.0)	
Solid tumor/brain tumor	105 (51.0)	35 (47.3)	70 (53.0)	
Weight (kg)	38.6 (21.2–52.1)	45.0 (36.2–56.7)	28.2 (18.6–48.4)	<0.001
Height (cm)	140.0 (119.0–157.0)	150.5 (138.5–160.8)	129.8 (111.0–152.0)	<0.001
BMI (kg/m^2^)	17.9 (15.6–22.3)	19.9 (17.3–24.6)	17.0 (15.1–20.5)	<0.001
Obese				0.025
Yes	65 (31.6)	31 (41.9)	34 (25.8)	
No	141 (68.4)	43 (58.1)	98 (74.2)	
Pubertal status				<0.001
Prepuberty	105 (51.0)	24 (32.4)	81 (61.4)	
Puberty	101 (49.0)	50 (67.6)	51 (38.6)	
Outdoor activities				<0.001
Yes	140 (68.0)	32 (43.2)	108 (81.8)	
No	66 (32.0)	42 (56.8)	24 (18.2)	
Duration of outdoor activities (hours/week)	3.0 (0–5.0)	0 (0–2.0)	4.0 (2.0–5.0)	<0.001
Dietary dairy intake (mL/week)	1250.0 (750.0–2400.0)	1000.0 (500.0–1237.5)	1500.0 (1000.0–3000.0)	<0.001
Steroids				0.949
Yes	101 (49.0)	37 (50.0)	64 (48.5)	
No	105 (51.0)	37 (50.0)	68 (51.5)	
Cumulative steroids (mg/m^2^)	6400 (4600–8400)	6400 (4600–8400)	6850 (4175–8400)	0.466
Surgery				0.341
Yes	94 (45.6)	30 (40.5)	64 (48.5)	
No	112 (54.4)	44 (59.5)	68 (51.5)	
Radiation				0.567
Yes	55 (26.7)	22 (29.7)	33 (25.0)	
No	151 (73.3)	52 (70.3)	99 (75.0)	
Intrathecal chemotherapy				0.212
Yes	87 (42.2)	36 (48.6)	51 (38.6)	
No	119 (57.8)	38 (51.4)	81 (61.4)	
Follow-up time (years)	2.3 (1.0–3.9)	2.3 (0.7–3.8)	2.4 (1.1–3.9)	0.744
**Laboratory Parameters**				
PTH (pg/mL)	41.2 (32.0–53.8)	47.4 (39.5–65.9)	38.0 (28.5–50.2)	<0.001
Calcium (mg/dL)	9.8 ± 0.4	9.7 ± 0.4	9.8 ± 0.4	0.009
Phosphorus (mg/dL)	4.5 ± 0.7	4.4 ± 0.8	4.5 ± 0.7	0.443
Alkaline phosphatase (U/L)	243.5 (171.5–304.0)	229.5 (127.2–341.2)	249.5 (193.8–301.2)	0.402
LDH (U/L)	224.0 (192.0–263.5)	211.0 (168.5–257.5)	234.0 (195.8–265.0)	0.045
ALT (U/L)	17.0 (13.0–24.0)	17.0 (12.0–25.0)	17.0 (13.0–23.0)	0.868
Albumin (g/dL)	4.6 (4.4–4.7)	4.5 (4.4–4.7)	4.6 (4.4–4.8)	0.156
Hb (g/dL)	13.0 (12.2–13.9)	13.3 (12.1–14.1)	12.9 (12.2–13.8)	0.406
Serum iron (μmol/L)	13.0 (9.1–16.3)	13.1 (9.7–17.2)	12.6 (8.9–16.1)	0.288
TIBC (μmol/L)	54.7 (49.0–60.1)	54.0 (47.9–60.9)	54.8 (49.2–59.8)	0.82
Transferrin saturation (%)	24.1 (16.2–30.1)	24.1 (16.4–33.2)	23.9 (15.9–29.4)	0.241
Ferritin (ng/mL)	79.5 (48.7–183.8)	89.6 (44.0–338.8)	77.0 (50.8–114.8)	0.156
Zinc (mg/L)	0.8 (0.7–0.9)	0.8 (0.7–0.9)	0.8 (0.7–0.9)	0.428
eGFR (mL/min/1.73 m^2^)	116.6 ± 28.0	117.8 ± 30.3	115.9 ± 26.7	0.632
eGFR				1
Decreased	36 (17.5)	13 (17.6)	23 (17.4)	
Normal	170 (82.5)	61 (82.4)	109 (82.6)	

^†^ Values are expressed as *n* (%), mean ± SD, or median (IQR). ALT, alanine aminotransferase; BMI, body mass index; eGFR, estimated glomerular filtration rate; Hb, hemoglobin; LDH, lactate dehydrogenase; PTH, parathyroid hormone; TIBC, total iron binding capacity.

**Table 3:** Multivariate analysis results showing independent risk factors for vitamin D deficiency in childhood cancer survivors.

Risk Factor	Crude OR (95% CI)	Adjusted OR (95% CI)	*p*-Value
Female sex	2.15 (1.20–3.85)	2.11 (1.08–4.13)	0.029
Obesity	2.08 (1.14–3.80)	2.01 (1.00–4.04)	0.05
Lack of outdoor activities	5.91 (3.12–11.2)	4.14 (2.08–8.21)	<0.001
Dietary dairy intake (mL/week)	0.51 (0.38–0.68)	0.59 (0.44–0.80)	<0.001

OR; odds ratio, CI; confidence interval.

## Data Availability

The datasets generated and analyzed during the current study are available from the corresponding author upon reasonable request.
